# Sexual and reproductive health behavior and unmet needs among a sample of adolescents living with HIV in Zambia: a cross-sectional study

**DOI:** 10.1186/s12978-018-0493-8

**Published:** 2018-03-27

**Authors:** Sumiyo Okawa, Sylvia Mwanza-Kabaghe, Mwiya Mwiya, Kimiyo Kikuchi, Masamine Jimba, Chipepo Kankasa, Naoko Ishikawa

**Affiliations:** 1 Department of Community and Global Health, Graduate School of Medicine, The University of Tokyo, Tokyo, Japan; 2 Department of Educational Psychology, Sociology, and Special Education, School of Education, University of Zambia, Lusaka, Zambia; 3 Paediatric HIV Centre of Excellence, University Teaching Hospital, Lusaka, Zambia; 4 Institute of Decision Science for a Sustainable Society, Kyushu University, Fukuoka, Japan; 5 Bureau of International Health Cooperation, National Center for Global Health and Medicine, Tokyo, Japan

**Keywords:** HIV, Adolescents, Sexual behavior, Sexual and reproductive health, Zambia

## Abstract

**Background:**

Adolescents living with HIV face challenges, such as disclosure of HIV status, adherence to antiretroviral therapy, mental health, and sexual and reproductive health (SRH). These challenges affect their future quality of life. However, little evidence is available on their sexual behaviors and SRH needs in Zambia. This study aimed at assessing their sexual behaviors and SRH needs and identifying factors associated with marriage concerns and a desire to have children.

**Methods:**

This cross-sectional study was conducted at the University Teaching Hospital from April to July 2014. We recruited 200 adolescents aged 15–19 years who were aware of their HIV-positive status. We collected data on their first and recent sexual behavior, concerns about marriage, and desire to have children. We used the Generalized Linear Model to identify factors associated with having concerns about marriage and desire to have children. We performed thematic analysis with open-ended data to determine their perceptions about marriage and having children in the future.

**Results:**

Out of 175 studied adolescents, 20.6% had experienced sexual intercourse, and only 44.4% used condoms during the first intercourse. Forty-eight percent had concerns about marriage, and 87.4% desired to have children. Marriage-related concerns were high among those who desired to have children (adjusted relative risk [ARR] = 2.51, 95% CI = 1.02 to 6.14). Adolescents who had completed secondary school were more likely to desire to have children (ARR = 1.35, 95% CI = 1.07 to 1.71). Adolescents who had lost both parents were less likely to want children (ARR = 0.80, 95% CI = 0.68 to 0.95). Thematic analysis identified that major concerns about future marriage were fear of disclosing HIV status to partners and risk of infecting partners and/or children. The reasons for their willingness to have children were the desire to be a parent, having children as family assets, a human right, and a source of love and happiness.

**Conclusions:**

Zambian adolescents living with HIV are at risk of engaging in risky sexual relationships and have difficulties in meeting needs of SRH. HIV care service must respond to a wide range of needs.

## Plain English summary

Adolescents living with HIV face difficulties including sexual and reproductive health (SRH). Physical growth and psychosocial development during adolescence could complicate the challenges. This study examined these adolescents’ sexual behaviors, needs, and concerns regarding SRH in Zambia.

This cross-sectional study was conducted at the University Teaching Hospital from April to July 2014. We collected data from 200 adolescents aged 15–19 years who were aware of their HIV-positive status and analyzed their sexual behavior and SRH needs with focus on whether they have concerns about marriage and desires for having children and the details of concerns and the reasons for desires.

Out of 175 adolescents, 20.6% had experienced sexual intercourse, 48.8% had concerns about their marriage, and 87.4% desired to have children. Adolescents who desired to have children were more likely to have concerns about marriage (ARR = 2.51). Adolescents who had completed secondary school were more likely to desire to have children (ARR = 1.35). Adolescents who had lost both parents were less likely to want children (ARR = 0.80). Major concerns about future marriage identified by the adolescents were fear of disclosing HIV status to partners and risk of infecting partners and/or children. The main reasons for their willingness to have children were the desire to be a parent, having children as family assets, a human right, and a source of love and happiness.

In conclusion, Zambian adolescents living with HIV have unmet needs regarding SRH. HIV care for adolescents should respond to a wide range of needs.

## Background

Human immunodeficiency virus (HIV) care and antiretroviral therapy (ART) services have significantly reduced AIDS-related deaths [1]. Adolescents are defined as those aged 10–19 years by the World Health Organization (WHO) [2]. They are one of the vulnerable populations affected by HIV. In 2015, 1.8 million adolescents were living with HIV, and this number is still growing globally [3]. About half of them contracted HIV infections from their mothers [4], and HIV is the second most common cause of death among them [5]. Even though they are living with HIV, they are healthier and live longer than those in previous decades. However, staying healthy remains a great challenge due to delayed diagnosis, limited access to treatment, adherence to life-long treatment, and chronic complications caused by treatment [6, 7].

Adolescents living with HIV face challenges such as recognition of HIV status, adherence to ART, mental health, and sexual and reproductive health (SRH) [8–11]. Rapid physical and psychosocial development throughout adolescence [5] could complicate such challenges. In addition, delayed puberty onset is commonly observed among perinatally-infected adolescents, which can result in low self-image, depression, and reproductive consequences [12]. For example, they make themselves targets for sexual abuse or they may easily engage in sexual intercourse to prove their worth. However, little evidence is available on sexual behaviors of adolescents living with HIV. In high-income counties, 27–46% of adolescents and young adults (ages 13–24) living with HIV have experienced sexual intercourse [13–16]. In low- and middle-income countries, it is less prevalent; for example, 25% of Ugandan adolescents and young adults (ages 11–21) and 5% of Thai adolescents (ages 11–18) living with HIV have experienced sexual intercourse [17, 18].

HIV infection affects primary needs of SRH during adolescence and adulthood. The WHO’s definitions of sexual health and reproductive health emphasize “having pleasurable and safe sexual experiences, free of coercion, discrimination and violence” [19] and “having the capability to reproduce and the freedom to decide if, when and how often to do so” [20]. Although they are basic human needs, it would be difficult for people living with HIV to meet these needs. Adolescents may experience various threats such as the risk of HIV transmission to partners, difficulties in disclosing HIV status to an intimate partner, managing subsequent rejection by partners, and optimal and consistent use of condoms [21, 22]. They may be particularly vulnerable to the risk of unsafe sexual activity due to peer pressure, poverty, stigma about HIV, ignorance by sexual partners, and alcohol consumption [17].

Despite multiple barriers to meeting their SRH needs, most adolescents living with HIV, at least in the United States, intended to have children [14]. Although few studies have assessed adolescents’ fertility intention in African countries, the rates of fertility intention among male and female adults were ranged between 37 and 51%, and the fertility intention was higher among those who were younger, had fewer children, were taking ART, and perceived themselves to have good health status [23–25]. However, the majority have not discussed their fertility intentions with health care workers [23, 25]. This implies that information and counselling on safe and timely pregnancy and childbirth are not widely provided to adolescents and adults living with HIV in African countries. The WHO has published two guidelines to promote client-friendly SRH services and respond to the complex needs of SRH and rights for adolescents living with HIV [2, 26]. However, little evidence is available to understand adolescents’ needs regarding intimate relationships, sexual behavior, marriage, and fertility intention in African countries.

The republic of Zambia has been affected by a high burden of HIV. In 2016, an estimated 12.4% of the adult population was living with HIV while 11,000 adolescents were newly infected [3]. The majority of young adolescents contracted HIV through mother-to-child transmission [27] whereas older adolescents are more likely to get HIV infection through sexual transmission or other routes [4]. Major adolescent health issues in the country are lack of knowledge about HIV, early initiation of sexual intercourse, sexually transmitted diseases, and teenage pregnancy [28, 29]. Qualitative studies have identified the difficulties of disclosing HIV status to a partner [30] and the unmet needs for information on HIV and SRH among adolescents living with HIV [31]. More than 870 health care facilities provided HIV care and treatment services across the country [32], and 67% of adults and 52% of children were receiving ART in 2016 [3]. The University Teaching Hospital is the central hospital in the capital city of Lusaka, which houses the Paediatric and Adult Centers of Excellence in HIV care and treatment. Although HIV care and treatment services have become widely available in the country, the existing services do not address challenges and needs of adolescents adequately. Particularly, limited evidence is available on sexual behaviors and SRH needs, and the quality of care in this area remains underdeveloped. We conducted this study to assess sexual behavior and SRH needs among adolescents living with HIV in Zambia and to identify factors associated with having concerns about marriage and desire to have children.

## Methods

### Study design and setting

We conducted this cross-sectional study at the Paediatric HIV Centre of Excellence and the Adult HIV Centre of Excellence at the University Teaching Hospital from April to July 2014. The two centres are the national referral centres and the model hospitals for adolescent HIV care and treatment services. The two centres equip various specialists and infrastructure to provide care, laboratory test, treatment, counselling, social welfare services, and support peer group activities. The Paediatric Centre provides the services to children and adolescents and transfers them to the Adult Centre when they turn 16 years old. The Adult Centre provides continuous care and treatment service to the adolescents. Health care workers routinely offer information on SRH to all the adolescents aged 10 years or older when they attend their routine clinical reviews. For adolescents who reported risky sexual behaviors during a regular clinical review, health care workers provided counselling services on the details of SRH and family planning methods, gave them condoms, and suggested that they go for a free counselling and testing service for HIV with their partners.

### Participants

The eligible study participants were adolescents aged 15–19 who were aware of their HIV status prior to the survey. Those aged 10–14 years were excluded from the study as SRH is a sensitive topic in Zambian culture, and it was deemed inappropriate to involve young adolescents in sexuality-related research.

We estimated a sample size of 165 to detect the association between educational experience and desire to have children based on a previous study that reported 55% prevalence of fertility intention among those with primary school education or higher and 32% among those with less than primary school education [25] using 5% of alpha, 80% of power, and 10% of potential missing data. However, we recruited 200 adolescents as the reference study used for the sample size estimation mainly targeted adults living with HIV. We did not use random sampling because reliable data of the target population and their contact information were not available during the study. We had a three-month data collection period to enroll at least 200 adolescents in the study as adolescent clients had review appointments every 3 months. We recruited the eligible adolescents when they came to the hospital for a clinical review appointment. Before recruiting adolescents, the research assistants asked health care workers or parents/primary caregivers to confirm the eligibility of the selected adolescents.

### Data collection

We used a self-administered structured questionnaire for data collection. The questionnaire included basic characteristics of the participating adolescents, their sexual behavior, SRH issues, concerns about future marriage, and desire for having children, based on existing literature [14, 21, 22, 30, 31]. We developed the questionnaire in English because the study sample used English daily. Before the survey, we conducted a pre-test of the questionnaire to assess their English literacy, edited the questionnaire using simple English, and confirmed that the majority could self-administer the questionnaire. We trained research assistants who were peer educators working for adolescents’ HIV care program at the study site. During the survey, respondents could ask the same-gender trained research assistants to help read-out in English or provide verbal translation into local language. Those who could not administer the questionnaire had an interview with the same-gender trained research assistants and responded everything except questions about sexual behavior.

Basic information included the adolescents’ gender, age, level of education, survival statuses of parents, and status of taking ART. Regarding sexual behaviors, we first asked whether an adolescent had ever had sexual intercourse. For those who had experienced sexual intercourse, we asked for details about the first sexual intercourse including their age, the partners’ age at that time, whether they had been aware of their HIV status, used a condom, and whether they were forced to have sexual intercourse. We also collected information on sexual experience in the preceding 12 months. For those who had had sexual intercourse during that period, we asked whether they disclosed their HIV status to their partner, knew their partners’ HIV status before the intercourse, used a condom, and ever asked their partner to take an HIV test. We also asked for information about SRH including whether they had disclosed their HIV status to their intimate partners, who they were comfortable talking to about SRH issues, and whether they had learned about prevention of HIV infection with an intimate partner, the risk of infecting an intimate partner with HIV, how to disclose HIV status to others, and how to develop intimate relationships.

The study outcomes were having concerns (or anxieties) about marriage and a desire to have children in the future. First, we provided a structured question “Do you have any concerns about marriage?” in the questionnaire, and then asked to administer their specific concerns with a single English sentence. In the same way, we asked another structured question “Do you want to have children in the future?” followed by asking their reasons for wanting children.

### Data analyses

For quantitative data, we first compared the distributions of background characteristics of the adolescents, their sexual behavior, and SRH issues using chi-square test. We then identified factors associated with having concerns about marriage and desire to have children by using the Generalized Linear Model. We included age, gender, education level, survival statuses of their parents, having someone to talk to about SRH issues, and having learned about four topics on SRH as independent variables of the models which were selected based on the existing literature [14, 23, 31, 33] and estimated crude and adjusted relative risks (RRs). We excluded missing data from the analyses and reported the number of respondents who declined to answer questions in the tables. We analyzed all the quantitative data using Stata (version 13.1).

For qualitative data, we performed thematic analysis to identify patterns of concerns about marriage and reasons they desired to have children. We followed the six phases in the data analysis process suggested by Braun [34]: (1) “familiarizing yourself with your data,” (2) “generating initial codes,” (3) “searching for themes,” (4) “reviewing themes,” (5) “defining and naming themes,” and (6) “producing the report.” Two authors (SO, KK) worked on Phases (1) and (2) independently and reviewed and discussed all codes to enhance reliability of the analyses. After that, the two authors conducted Phases (3), (4), and (5). Finally, SO conducted Phase (6). We manually analyzed all the qualitative data.

### Ethical considerations

We obtained ethical approval from the Biomedical Research Ethics Committee of the University of Zambia and the Institutional Ethics Committee of National Center for Global Health and Medicine, Japan. We obtained written informed consent from all participants and written assent from parents or primary caregivers of adolescents aged 15, 16, and 17 years. We anticipated that adolescents may feel pressured to participate in the study as we recruited them at the hospital where they were receiving care. In addition, they may hesitate to respond about sexual behavior if they suspect the individual data could be shared with the hospital staff. To address these potential issues, we informed each adolescent about voluntary participation without any harm if they avail of care in the future and confidentiality in data management.

## Results

We recruited 200 eligible adolescents in the study. We excluded two adolescents who withdrew during the survey and 15 adolescents who did not respond about their sexual behavior due to limited English literacy or voluntarily declined to answer. We excluded eight from the dataset because they were outside of the eligible criteria for age, although we asked their age at recruitment. Finally, we included the data of 175 adolescents in the analysis. Seventy-two (41.1%) were boys, and 103 (58.9%) were girls. Sixty-five percent had lost their fathers and/or mothers, and 94.3% were undergoing ART. No significant gender differences were found in the distributions of the basic characteristics (Table 1).

**Table 1:** Basic characteristics of participants (*n* = 175)

	Total		Boys		Girls		
	*n* = 175		*n* = 72		*n* = 103		
	*n*	(%)	*n*	(%)	*n*	(%)	*p*
Age							
15 years	23	(13.1)	7	(9.7)	16	(15.5)	0.28
16 years	42	(24.0)	23	(31.9)	19	(18.5)	
17 years	38	(21.7)	13	(18.1)	25	(24.3)	
18 years	42	(24.0)	17	(23.6)	25	(24.3)	
19 years	30	(17.1)	12	(16.7)	18	(17.5)	
Educational experience							
Incomplete primary school	26	(14.9)	11	(15.3)	15	(14.6)	0.93
Completed primary school	88	(50.3)	35	(48.6)	53	(51.1)	
Completed secondary school or higher	61	(34.9)	26	(36.1)	35	(34.0)	
Parental survival status							
Both parents are alive	61	(34.9)	30	(41.7)	31	(30.1)	0.09
Mother was dead	34	(19.4)	13	(18.1)	21	(20.4)	
Father was dead	35	(20.0)	17	(23.6)	18	(17.5)	
Both parents were dead	45	(25.7)	12	(16.7)	33	(32.0)	
Currently taking ART							
Yes	165	(94.3)	70	(97.2)	95	(92.2)	0.16
No	10	(5.7)	2	(2.8)	8	(7.8)	

Table 2 shows the sexual behaviors of the adolescents. Thirty-six adolescents (20.6%) had ever experienced sexual intercourse with a mean age of 16.2 (SD 1.0) years among boys and 15.7 (SD 2.0) years among girls at the first intercourse (*p* = .51). Girls were more likely to have an older-aged partner than were boys (*p* < .01). Although 55.6% were already aware of their HIV status at the first intercourse, at least 33.3% did not use a condom. Only girls (*n* = 4) reported that their first sexual intercourse was forced. In the last 12 months prior to the survey, 22 adolescents (12.6%) reported having had sexual intercourse. Of the 22 adolescents, girls were more likely to disclose their HIV status to their partners (*p* = .01) and to know their partner’s HIV status (*p* = .05) before engaging in the sexual relationship.

**Table 2:** Sexual behavior (*n* = 175)

	Total		Boys		Girls		
	*n*	(%)	*n*	(%)	*n*	(%)	*p*
First sexual experience							
Ever had sexual intercourse							
Yes	36	(20.6)	15	(20.8)	21	(20.4)	0.90
No	133	(76.0)	54	(75.0)	79	(76.7)	
Declined to answer	6	(3.4)	3	(4.2)	3	(2.9)	
Age at the first sexual intercourse (*n* = 21)	16.0	(1.6)	16.2	(1.0)	15.7	(2.0)	0.51
Partner’s age at the first sexual intercourse (*n* = 19)	18.4	(3.0)	16.2	(2.6)	20.4	(1.8)	< 0.01
Already aware of HIV status (*n* = 36)							
Yes	20	(55.6)	6	(40.0)	14	(66.7)	0.19
No	11	(30.6)	7	(46.7)	4	(19.1)	
Declined to answer	5	(13.9)	2	(13.3)	3	(14.3)	
Used condom at first sexual intercourse (*n* = 36)							
Yes	16	(44.4)	6	(40.0)	10	(47.6)	0.77
No	12	(33.3)	6	(40.0)	6	(28.6)	
Declined to answer	8	(22.2)	3	(20.0)	5	(23.8)	
First sexual intercourse was forced (*n* = 36)							
Yes	4	(11.1)	0	(0.0)	4	(19.1)	0.17
No	25	(69.4)	11	(73.3)	14	(66.7)	
Declined to answer	7	(19.4)	4	(26.7)	3	(14.3)	
Sexual experience in the last 12 months							
Had sexual intercourse (*n* = 175)							
Yes	22	(12.6)	7	(9.7)	15	(14.6)	0.49
No	149	(85.1)	64	(88.9)	85	(82.5)	
Declined to answer	4	(2.3)	1	(1.4)	3	(2.9)	
Disclosed HIV status to partner in advance (*n* = 22)							
Yes	11	(50.0)	1	(14.3)	10	(66.7)	0.01
No	8	(36.4)	3	(42.9)	5	(33.3)	
Declined to answer	3	(13.6)	3	(42.9)	0	(0.0)	
Already knew partner’s HIV status (*n* = 22)							
Yes	10	(45.5)	1	(14.3)	9	(60.0)	0.05
No	12	(54.6)	6	(85.7)	6	(40.0)	
Used condom (*n* = 22)							
Yes	12	(54.6)	5	(71.4)	7	(46.7)	0.53
No	6	(27.3)	1	(14.3)	5	(33.3)	
Declined to answer	4	(18.2)	1	(14.3)	3	(20.0)	
Asked partner for HIV testing (*n* = 22)							
Yes	7	(31.8)	1	(14.3)	6	(40.0)	0.38
No	9	(40.9)	3	(42.9)	6	(40.0)	
Declined to answer	6	(27.3)	3	(42.9)	3	(20.0)	

Table 3 shows the SRH issues of the adolescents. About half (48.8%) reported that they had concerns about marriage, and 87.4% desired to have children in the future with no significant gender differences. Twenty percent had disclosed their HIV status to their partners at least once. Higher proportions of the adolescents had learned about the prevention of HIV infection to a partner (82.9%) and the risk of HIV transmission to a partner (74.3%) compared with those who had learned about how to disclose their HIV status to others (58.9%) and how to develop intimate relationships (49.7%).

**Table 3:** Sexual and reproductive health needs

	Total		Boys		Girls		
	*n*	(%)	*n*	(%)	*n*	(%)	*p*
Have concerns about marriage (*n* = 168)							
Yes	82	(48.8)	31	(45.6)	51	(51.0)	0.49
No	86	(51.2)	37	(54.4)	49	(49.0)	
Desire to have children (*n* = 175)							
Yes	153	(87.4)	65	(90.3)	88	(85.4)	0.34
No	22	(12.6)	7	(9.7)	15	(14.6)	
Ever disclosed HIV status to an intimate partner (*n* = 172)							
Yes	35	(20.4)	14	(19.7)	21	(20.8)	0.48
No	71	(41.3)	33	(46.5)	38	(37.6)	
Never had an intimate partner	66	(38.4)	24	(33.8)	42	(41.6)	
Person to talk about SRH issues							
Friend	32	(18.3)	8	(11.1)	24	(23.3)	0.31
Mother	29	(16.6)	12	(16.7)	17	(16.5)	
Peers living with HIV	17	(9.7)	7	(9.7)	10	(9.7)	
Health care worker	7	(4.0)	2	(2.8)	5	(4.9)	
Other	64	(36.6)	32	(44.4)	32	(31.1)	
Nobody	26	(14.9)	11	(15.3)	15	(14.6)	
Education topics on HIV and SRH ever learned							
Preventing HIV transmission to partner							
Yes	145	(82.9)	60	(83.3)	85	(82.5)	0.89
No	30	(17.1)	12	(16.7)	18	(17.5)	
Risk of HIV transmission to partner							
Yes	130	(74.3)	52	(72.2)	78	(75.7)	0.60
No	45	(25.7)	20	(27.8)	25	(24.3)	
How to disclose HIV status to others							
Yes	103	(58.9)	40	(55.6)	63	(61.2)	0.46
No	72	(41.1)	32	(44.4)	40	(38.8)	
How to develop intimate relationships							
Yes	87	(49.7)	42	(58.3)	45	(43.7)	0.06
No	88	(50.3)	30	(41.7)	58	(56.3)	

Table 4 shows the factors associated with having concerns about marriage (*n* = 168). Adolescents who wanted to have their own children were more likely to have concerns about their future marriage compared with those who did not desire to have children (Adjusted RR [ARR] = 2.51, 95% CI = 1.02 to 6.14). Table 5 shows the factors associated with desiring to have children (*n* = 175). The adolescents who had completed secondary school or higher were more likely to desire to have children than those who had not completed primary school (ARR = 1.35, 95% CI = 1.07 to 1.71). Adolescents whose parents were both dead were less likely to desire to have children compared with those whose parents were both alive (ARR = 0.80, 95% CI = 0.68 to 0.95).

**Table 4:** Factors associated with having concerns about marriage (*n* = 168)

	cRR	(95%CI)	aRR	(95%CI)
Age	0.99	(0.88–1.12)	1.11	(0.96–1.29)
Gender				
Boy	0.89	(0.65–1.24)	0.85	(0.62–1.15)
Girl	1.00		1.00	
Educational experience				
Incomplete primary school	1.00		1.00	
Completed primary school	1.55	(0.89–2.71)	1.50	(0.86–2.60)
Completed secondary school or higher	1.22	(0.67–2.23)	0.97	(0.51–1.83)
Parental survival status				
Both parents are alive	1.00		1.00	
Mother was dead	0.85	(0.55–1.32)	0.82	(0.53–1.27)
Father was dead	0.91	(0.60–1.37)	0.93	(0.64–1.35)
Both parents were dead	0.74	(0.48–1.13)	0.73	(0.48–1.12)
Have someone to talk about SRH issues				
Yes	1.20	(0.73–1.98)	1.25	(0.78–2.01)
No	1.00		1.00	
Learned about HIV and SRH				
0–3 items	1.00		1.00	
4 items	0.78	(0.55–1.12)	0.80	(0.55–1.17)
Want to have my children				
Yes	2.64	(1.08–6.43)	2.51	(1.02–6.14)
No	1.00		1.00	

Age is a continuous variable. cRR and aRR mean the crude and adjusted relative risk

**Table 5:** Factors associated with desiring to have children (*n* = 175)

	cRR	95%CI	aRR	95%CI
Age	1.01	(0.97–1.05)	0.97	(0.92–1.03)
Gender				
Boy	1.06	(0.95–1.18)	1.02	(0.92–1.14)
Girl	1.00		1.00	
Educational experience				
Incomplete primary school	1.00		1.00	
Completed primary school	1.11	(0.88–1.39)	1.17	(0.94–1.45)
Completed secondary school or higher	1.24	(0.99–1.54)	1.35	(1.07–1.71)
Parental survival status				
Both parents are alive	1.00		1.00	
Mother was dead	0.90	(0.77–1.04)	0.88	(0.76–1.03)
Father was dead	0.93	(0.82–1.06)	0.92	(0.81–1.05)
Both parents were dead	0.82	(0.69–0.97)	0.80	(0.68–0.95)
Have someone to talk about SRH issues				
Yes	1.10	(0.90–1.34)	1.10	(0.90–1.34)
No	1.00		1.00	
Learned about HIV and SRH				
0–3 items	1.00		1.00	
4 items	1.02	(0.90–1.14)	1.00	(0.88–1.15)

Age is a continuous variable. cRR and aRR mean the crude and adjusted relative risk

Table 6 shows the adolescents’ concerns or anxieties about future marriage. Out of 82 adolescents who reported that they were concerned about future marriage (Table 3), 63 (76.8%) described the specific concerns. The four major themes that emerged were: disclosing HIV status to partner, options in marriage, HIV transmission to partner and children. Adolescents were anxious about how to disclose HIV status to their intimate partners and their reactions afterwards: *“I wouldn’t know how to tell him my HIV status if he is not HIV infected”* (Girl, age 15); *“If the person who will want to marry me finds out that I am HIV positive, he will change his mind about marrying me*” (Girl, age 17). Adolescents also worried about the risk of HIV transmission to their partners and children: *“Will I have to risk her health just for a child? If not, will she have to carry the burden with me?”* (Boy, age 19); *“Is there medicine that can reduce the risk of the baby contracting HIV from the mother?*” (Girl, age 17). To avoid infecting partner and children, some adolescents also considered remaining single or marrying a partner also living with HIV: *“I sometimes think it's better to remain single forever*” (Girl, age 18); “*I think I will have a wife who is (HIV) positive”* (Boy, age 17).

**Table 6:** Concerns about future marriage

Main theme	Sub-theme
Disclosing HIV status to partner	Do not know how to disclose my HIV status to partner
	Concern about partner'’s reaction after disclosing my HIV status
Options in marriage	Not getting married may be better
	HIV-positive partner may be suitable
HIV transmission to partner	Don'’t know how to protect partner from HIV infection
	Concern about the risk of HIV transmission to partner
HIV transmission to children	Don'’t know how to protect children from HIV infection
	Concern about the risk of HIV transmission to children

Out of 82 adolescents who had concerns about future marriage, 63 (76.8%) provided open-ended answers to explain the specific concerns or anxieties

Table 7 shows the reasons why adolescents desired to have children. Out of 153 adolescents who showed the desires to have children in Tables 3, 102 (66.7%) explained the reasons. From the thematic analysis, four major reasons emerged: desire for parenting, children as family assets, positive perception on children, and natural matter. Some adolescents wished to become a parent: “*I want to know how it feels to be a mother”* (Girl, age 18). They expected to have children as household assets: *“I want them to help me when I get old”* (Girl, age 15); “*I want my family name to continue”* (Boy, age 17). Some adolescents showed their love for children and regarded children as sources of happiness and joy in their lives: “*A child will bring happiness to me and my family”* (Girl, age 17). They also saw children as a blessing: *“Children all are wonderful gifts from God”* (Girl, age 19). Some adolescents stated that having children is a natural outcome of marriage and a human right: *“I am a human being and have the right to have a child. My (HIV-positive) status does not matter”* (Girl, age 17).

**Table 7:** Reasons that adolescents desire to have children

Main theme	Sub-theme
Desire for parenting	Desire for becoming a parent
	Desire for taking care of children
Children as family assets	Children as my caretakers
	Children as the successors of family
Positive perception on children	Love for children
	Happiness brought by children
	Blessing from God
Natural matter	Natural outcome of marriage
	Human right

Out of 153 adolescents who desired to have children in the future, 102 (66.7%) explained the reasons

## Discussion

Twenty percent of the adolescents in our sample had experienced sexual intercourse, and at least 30% of them did not use a condom during the first intercourse. Nearly half of the adolescents expressed concerns about their future marriage. In contrast, the majority of them desired to have their own children in the future. Those who desired to have children were more likely to worry about their marriage. To the best of our knowledge, this is the first quantitative evidence built on the existing study findings in Zambia [30, 31].

Adolescents in the study were less likely to have had sexual intercourse (20.6%) compared with the adolescents (ages 15–19) in the national representative data (49.1% among girls and 47.7% among boys) [29]. Similarly, in the United States, perinatally HIV-infected youths were less likely to practice sexual intercourse compared with uninfected youths [35]. However, it should be highlighted that sexually active adolescents in this study engaged in risky sexual intercourse; nearly 30% did not use a condom during their first intercourse and recent intercourse. Moreover, sexual intercourse commonly took place without recognizing the HIV status, particularly among HIV-positive boys and their partners. In addition, girls had relatively older partners and tended to have forced intercourse. This indicates that girls could be particularly vulnerable to control by their partners’ demands for unprotected intercourse. Both girls and boys were sexually active, but the process or context of engaging in sexual relationships was not the same.

Adolescents’ concerns about marriage were not related only to marriage. Their concerns were widely associated with intimate relationships—namely, disclosing their HIV status to partners, potential rejection afterwards, and the risk of HIV transmission. This indicates that adolescents were facing a dual pressure with rejection and infection in intimate relationships. Similar findings have been reported in Canada, the United States, and Rwanda [21, 22, 36]. Most adolescents in this study had learned about the risk of HIV transmission and the preventive methods, but they had limited information on the ways to develop intimate partnerships and disclose HIV status to others. In addition, health care workers were less utilized as primary counsellors or advisors for their SRH issues. Health care workers should make further effort to develop a better relationship with adolescents and provide them with comprehensive information on SRH.

Most of the adolescents expressed the desire to have children. They felt that having children has an important meaning in their adulthood. It means that they can achieve their dreams and happiness, secure family assets, and that it is human nature and their right. On the other hand, they were afraid of HIV transmission to their children and had insufficient knowledge about prevention of mother-to-child transmission. Similarly, in the United States and Rwanda, a strong fear of mother-to-child transmission was associated with lower intention to have children [14, 36]. In this study, adolescents who had lost both parents were less likely to desire to have their own children. Loss of parents is linked to multiple negative consequences: psychological distress, caretaking responsibility for younger siblings, dropping out of school, and vulnerability to sexually transmitted diseases and early pregnancy [33, 37, 38]. Due to such childhood experiences and uncertain life expectancy due to being affected by HIV, orphaned adolescents may have a lower motivation to have their own children. Adolescents who completed secondary education had a higher intention to have children. Educational experience is known as a protective factor against health risks in adolescence [39]. On the other hand, those who did not complete primary education had a passive desire for having children. This may indicate that they had limited access to information and/or low literacy about SRH. Orphaned or low-educated adolescents need special support for their proactive choice of future pregnancy and childbirth.

Adolescents showed unmet SRH needs about intimate and sexual relationships, marriage, pregnancy, and childbirth. However, the current HIV care and treatment services for adolescents do not fully respond to their needs. The health care workers must make more efforts to reach all adolescents regardless of their gender, socio-economic status (e.g. family structure, educational level), and childhood background. Education and counselling should primarily emphasize basic prevention of HIV transmission for safe sexual relationships while considering gender sensitivity and vulnerability. In addition, information on the benefits and risks of disclosing HIV status to a partner should be provided. Follow-up counselling after self-disclosure is also essential for those in psychological distress facing negative outcomes of disclosure. To respond to the complicated SRH needs of adolescents, training on adolescent-centered SRH care should be offered to health care workers. In addition, many Zambians experience the first marriage and pregnancy in late adolescence or early adulthood. It will be beneficial for adolescents to discuss and prepare for safe pregnancy and childbirth in collaboration with family planning and prevention of mother-to-child transmission of HIV programs.

This study has several limitations. First, the generalizability of the study findings is limited as we conducted the study at a tertiary hospital in the national capital with no comparison groups. Second, convenience sampling for recruiting study participants at regular clinical reviews may have contributed to selection bias, because adolescents with no access or no compliance with care were not included in the study, but they would have different patterns of sexual behaviors and different needs regarding SRH. Third, we did not collect data on marital status or route of HIV transmission, which could influence to sexual behavior. Further studies at multiple sites with various target populations would be recommended, and sub-group analysis by geographical area, compliance with care, marital status, and the routes of HIV transmission could provide the study results more clearly. Third, we collected information on sensitive issues about sexual behaviors, which may lead to under-reporting and an increase in missing data. Thus, the study findings could be under-estimated and should not be simply compared with results in other settings. The future study needs to use anonymous survey methods, such as computer-assisted survey. Fourth, the small sample size may limit the power to identify significant associations between participants’ characteristics and the study outcome. Despite the limitations, this study used quantitative and qualitative data and identified the knowledge gaps in how HIV infection affects current and future SRH and well-being among adolescents.

## Conclusions

Zambian adolescents living with HIV were sexually active. In addition, they were highly concerned about future marriage and fertility opportunities due to potential refusal by their partners and the risks of HIV transmission to their partners and children. HIV care and treatment services should cover a wider area of SRH needs and reach all adolescents regardless of their socio-demographic and -economic backgrounds.

## Abbreviations

ART: Antiretroviral therapy
HIV: Human immunodeficiency virus
SRH: Sexual and reproductive health
WHO: World Health Organization

## References

[1] UNAIDS (2014). The gap report.

[2] WHO (2013). HIV and adolescents: guidance for HIV testing and counselling and care for adolescents living with HIV: recommendations for a public health approach and considerations for policy-makers and managers.

[3] UNAIDS. AIDSinfo. No date. http://aidsinfo.unaids.org/#. Accessed 29 Sep 2017.

[4] UNICEF (2014). 2014 annual results report: HIV and AIDS.

[5] WHO (2014). Health for the world's adolescents: a second chance in the second decade.

[6] Ferrand RA, Munaiwa L, Matsekete J, Bandason T, Nathoo K, Ndhlovu CE (2010). Undiagnosed HIV infection among adolescents seeking primary health care in Zimbabwe. Clin Infect Dis.

[7] Mofenson LM, Cotton MF (2013). The challenges of success: adolescents with perinatal HIV infection. J Int AIDS Soc.

[8] Gray GE (2010). Adolescent HIV--cause for concern in southern Africa. PLoS Med.

[9] Koenig LJ, Nesheim S, Abramowitz S (2011). Adolescents with perinatally acquired HIV: emerging behavioral and health needs for long-term survivors. Curr Opin Obstet Gynecol.

[10] Agwu AL, Fairlie L (2013). Antiretroviral treatment, management challenges and outcomes in perinatally HIV-infected adolescents. J Int AIDS Soc.

[11] Mellins CA, Malee KM (2013). Understanding the mental health of youth living with perinatal HIV infection: lessons learned and current challenges. J Int AIDS Soc.

[12] Williams PL, Abzug MJ, Jacobson DL, Wang J, Van Dyke RB, Hazra R (2013). Pubertal onset in children with perinatal HIV infection in the era of combination antiretroviral treatment. AIDS.

[13] Koenig LJ, Pals SL, Chandwani S, Hodge K, Abramowitz S, Barnes W (2010). Sexual transmission risk behavior of adolescents with HIV acquired perinatally or through risky behaviors. J Acquir Immune Defic Syndr.

[14] Ezeanolue EE, Wodi AP, Patel R, Dieudonne A, Oleske JM (2006). Sexual behaviors and procreational intentions of adolescents and young adults with perinatally acquired human immunodeficiency virus infection: experience of an urban tertiary center. J Adolesc Health.

[15] Wiener LS, Battles HB, Wood LV (2007). A longitudinal study of adolescents with perinatally or transfusion acquired HIV infection: sexual knowledge, risk reduction self-efficacy and sexual behavior. AIDS Behav.

[16] Brogly SB, Watts DH, Ylitalo N, Franco EL, Seage GR, Oleske J (2007). Reproductive health of adolescent girls perinatally infected with HIV. Am J Public Health.

[17] Bakeera-Kitaka S, Nabukeera-Barungi N, Nostlinger C, Addy K, Colebunders R (2008). Sexual risk reduction needs of adolescents living with HIV in a clinical care setting. AIDS Care.

[18] Lolekha R, Boon-Yasidhi V, Leowsrisook P, Naiwatanakul T, Durier Y, Nuchanard W (2015). Knowledge, attitudes, and practices regarding antiretroviral management, reproductive health, sexually transmitted infections, and sexual risk behavior among perinatally HIV-infected youth in Thailand. AIDS Care.

[19] WHO (2017). Sexual health.

[20] WHO (2017). Reproductive health.

[21] Fernet M, Wong K, Richard ME, Otis J, Levy JJ, Lapointe N (2011). Romantic relationships and sexual activities of the first generation of youth living with HIV since birth. AIDS Care.

[22] Fair C, Albright J (2012). "Don't tell him you have HIV unless he's 'the one'": romantic relationships among adolescents and young adults with perinatal HIV infection. AIDS Patient Care STDs.

[23] Cooper D, Moodley J, Zweigenthal V, Bekker LG, Shah I, Myer L (2009). Fertility intentions and reproductive health care needs of people living with HIV in cape town, South Africa: implications for integrating reproductive health and HIV care services. AIDS Behav.

[24] Mmbaga EJ, Leyna GH, Ezekiel MJ, Kakoko DC (2013). Fertility desire and intention of people living with HIV/AIDS in Tanzania: a call for restructuring care and treatment services. BMC Public HealthBMC Public Health..

[25] Kawale P, Mindry D, Stramotas S, Chilikoh P, Phoya A, Henry K (2014). Factors associated with desire for children among HIV-infected women and men: a quantitative and qualitative analysis from Malawi and implications for the delivery of safer conception counseling. AIDS Care..

[26] WHO (2017). Consolidated guideline on sexual and reproductive health and rights of women living with HIV.

[27] Menon A, Glazebrook C, Campain N, Ngoma M (2007). Mental health and disclosure of HIV status in Zambian adolescents with HIV infection: implications for peer-support programs. J Acquir Immune Defic Syndr..

[28] National AIDS Council, Ministry of Health, Ministry of Community Development Mother and Child Health (2014). Zambia country report: monitoring the declaration of commitment on HIV and AIDS and the Universal access: biennial report.

[29] Central Statistical Office (CSO) [Zambia], Ministry of Health (MOH) [Zambia], ICF International (2014). Zambia Demographic and Health Survey 2013–-14.

[30] Mburu G, Hodgson I, Kalibala S, Haamujompa C, Cataldo F, Lowenthal ED (2014). Adolescent HIV disclosure in Zambia: barriers, facilitators and outcomes. J Int AIDS Soc..

[31] Hodgson I, Ross J, Haamujompa C, Gitau-Mburu D (2012). Living as an adolescent with HIV in Zambia -- lived experiences, sexual health and reproductive needs. AIDS Care..

[32] National HIV, AIDS STI, TB Council (2017). National HIV AIDS strategic framework 2017–-2021.

[33] Cluver LD, Orkin M, Gardner F, Boyes ME (2012). Persisting mental health problems among AIDS-orphaned children in South Africa. J Child Psychol Psychiatry..

[34] Braun V, Clarke V (2006). Using thematic analysis in Psychology. Qual Res Psychol..

[35] Elkington KS, Bauermeister JA, Robbins RN, Gromadzka O, Abrams EJ, Wiznia A (2012). Individual and contextual factors of sexual risk behavior in youth perinatally infected with HIV. AIDS Patient Care STDS..

[36] Van Nuil JI, Mutwa P, Asiimwe-Kateera B, Kestelyn E, Vyankandondera J, Pool R (2014). "Let's talk about sex": a qualitative study of Rwandan adolescents' views on sex and HIV. PLoS One.

[37] Kang M, Dunbar M, Laver S, Padian N (2008). Maternal versus paternal orphans and HIV/STI risk among adolescent girls in Zimbabwe. AIDS Care..

[38] Bazile J, Rigodon J, Berman L, Boulanger VM, Maistrellis E, Kausiwa P (2015). Intergenerational impacts of maternal mortality: Qualitative findings from rural Malawi. Reprod Health.

[39] Viner RM, Ozer EM, Denny S, Marmot M, Resnick M, Fatusi A (2012). Adolescence and the social determinants of health. Lancet..

